# Association between B Vitamins Supplementation and Risk of Cardiovascular Outcomes: A Cumulative Meta-Analysis of Randomized Controlled Trials

**DOI:** 10.1371/journal.pone.0107060

**Published:** 2014-09-19

**Authors:** Chi Zhang, Zhi-Yong Wang, Ying-Yi Qin, Fei-Fei Yu, Yu-Hao Zhou

**Affiliations:** 1 Department of Neurosurgery, Shanghai Seventh People's Hospital, Shanghai, China; 2 Department of Information, Changhai Hospital, Second Military Medical University, Shanghai, China; 3 Department of Health Statistics, Second Military Medical University, Shanghai, China; 4 Department of Rehabilitation Institute, Shanghai Seventh People's Hospital, Shanghai, China; Scuola Superiore Sant'Anna, Italy

## Abstract

**Background:**

Observational studies suggest that B vitamin supplementation reduces cardiovascular risk in adults, but this association remains controversial. This study aimed to summarize the evidence from randomized controlled trials (RCTs) investigating B vitamin supplementation for the primary or secondary prevention of major adverse cardiovascular outcomes and to perform a cumulative meta-analysis to determine the evidence base.

**Methodology and Principal Findings:**

In April 2013, we searched PubMed, Embase, and the Cochrane Library to identify relevant RCTs. We included RCTs investigating the effect of B vitamin supplementation on cardiovascular outcome. Relative risk (RR) was used to measure the effect using a random-effect model. Statistical heterogeneity scores were assessed using the Q statistic. We included data on 57,952 individuals from 24 RCTs: 12 primary prevention trials and 12 secondary prevention trials. In 23 of these trials, 10,917 major adverse cardiovascular events (MACE) occurred; in 20 trials, 7,203 deaths occurred; in 15 trials, 3,422 cardiac deaths occurred; in 19 trials, 3,623 myocardial infarctions (MI) occurred; and in 18 trials, 2,465 strokes occurred. B vitamin supplementation had little or no effect on the incidence of MACE (RR, 0.98; 95% confidence interval [CI]: 0.93–1.03; P = 0.37), total mortality (RR, 1.01; 95% CI: 0.97–1.05; P = 0.77), cardiac death (RR, 0.96; 95% CI: 0.90–1.02; P = 0.21), MI (RR, 0.99; 95% CI: 0.93–1.06; P = 0.82), or stroke (RR, 0.94; 95% CI: 0.85–1.03; P = 0.18).

**Conclusion/Significance:**

B vitamin supplementation, when used for primary or secondary prevention, is not associated with a reduction in MACE, total mortality, cardiac death, MI, or stroke.

## Introduction

The potential role of B vitamins (folate, B6, and B12) in reducing cardiovascular risk has been supported by observational studies [1]–[5]. Although the mechanism of action is unclear, B vitamins may affect cardiovascular outcome by lowering homocysteine concentrations, which correlate strongly with the risk of coronary disease [1]–[5] and stroke [6]–[8]. A meta-analysis [9] of observational studies suggested that lowering the plasma homocysteine level by 25% reduced the risk of coronary heart disease by 11% and the risk of stroke by 19%. Daily supplementation with folic acid has been shown to lower the plasma homocysteine level by approximately 25% and adding vitamin B12 further lowers the level by approximately 7%, indicating that B vitamins supplements lower homocysteine levels significantly [10]–[11]. Despite its ability to lower homocysteine levels, meta-analyses of randomized controlled trials (RCTs) [12]–[19] indicate that, although folic acid supplementation may reduce the risk of stroke in specific subsets of patients, it is not associated with a reduction in a composite of all-cause death, nonfatal acute myocardial infarction (MI), acute hospitalization for unstable angina pectoris, and nonfatal thromboembolic stroke (MACE), MI, cardiac death, or total mortality. The association between B vitamin supplementation and reduction of cardiovascular risk has not been confirmed by a RCT [20]–[31]. Furthermore, previous meta-analyses have not investigated the potential interaction of supplementation with both vitamin B6 and B12 on cardiovascular risk [12]–[17], [19].

The effect of B vitamin supplementation on primary and secondary prevention of adverse cardiovascular outcomes has been studied in numerous RCTs [20]–[43]. In this study, we performed a meta-analysis of these RCTs to evaluate the effect of B vitamin supplementation on cardiovascular risk in specific subpopulations and attempt to determine the role of folic acid supplementation interaction with vitamin B6 and B12 in reducing cardiovascular risk. Furthermore, we used cumulative meta-analysis to determine the evidence base for routine B vitamin supplementation in clinical practice.

## Methods

### Data Sources, Search Strategy, and Selection Criteria

This review was conducted and reported according to the Preferred Reporting Items for Systematic Reviews and Meta-Analysis Statement, 2009 (Checklist S1) [44]. RCTs evaluating the effect of B vitamin supplementation on cardiovascular risk written in the English language were included in our study, regardless of the publication status (published, in press, or in progress) and the effect of B vitamin supplementation on MACE, total mortality, cardiac death, MI, and stroke were examined. Relevant trials were identified using the following procedure:

(1) Electronic searches: we searched PubMed, Embase, and the Cochrane Central Register of Controlled Trials electronic databases for articles published through April 2013, using “B vitamins” AND “randomized controlled trials” AND “clinical trials” AND “human” AND “English” as the search terms.

(2) Other sources: we searched ongoing RCTs in the metaRegister of Controlled Trials, which lists trials that are registered as completed but not yet published. Furthermore, we reviewed bibliographies of publications for potentially relevant trials. Medical subject headings, methods, patient population, interventions, and outcome variables of these studies were used to identify relevant trials.

The literature search, data extraction, and quality assessment were undertaken by 2 investigators (CZ and PJM) independently with a standardized approach. Any inconsistencies between these investigators were identified by the primary investigator (YHZ) and resolved by consensus. We restricted our study to RCTs, which are less likely than observational studies to be subject to confounding variables or bias. A study was eligible for inclusion in our meta-analysis if the following criteria were met: (1) the study was a RCT; (2) the trial evaluated the effects of B vitamin supplementation compared with placebo; (3) the study's follow-up continued for at least 6 months; and (4) the trial reported at least 1 of the following outcomes: MACE, total mortality, cardiac death, MI, or stroke.

### Data Collection and Quality Assessment

All data from included trials was extracted independently by 2 investigators (CZ and PJM) using a standardized protocol. Each data set was reviewed by a third investigator (YHZ), and any discrepancies between the 2 investigator's data were resolved by discussion. The data collected from each study included first author or study group name, publication year, study design, type of blinding, number of patients, percentage of men, mean age, background fortification, current disease status, baseline total homocysteine level, baseline folate level, intervention regimens, controls, and the duration of follow-up. The outcomes investigated included MACE, total mortality, cardiac death, MI, and stroke. Study quality was assessed using the Jadad score [45], which is based on the 5 following subscales: randomization (1 or 0), concealment of the treatment allocation (1 or 0), blinding (1 or 0), completeness of follow-up (1 or 0), and the use of intention-to-treat analysis (1 or 0). A score system ranging from 1 to 5 has been developed for quality assessment.

### Statistical Analysis

We assigned the results of each RCT as dichotomous frequency data. Individual study relative risks (RR) and 95% confidence intervals (CI) were calculated from event numbers extracted from each trial before data pooling. The overall RR and 95% CI of MACE, total mortality, cardiac death, MI, and stroke were also calculated. Both fixed-effect and random-effect models were used to evaluate the pooled RR for B vitamin supplementation compared with placebo. Although both models yielded similar findings, results from the random-effect model, which assume that the true underlying effect varies among included trials, are presented here [46]–[47]. Heterogeneity of the treatment effect between studies was evaluated using the Q statistic, and P values <0.10 were considered statistically significant [48]–[49]. In the cumulative meta-analysis, outcome data for MACE, total mortality, cardiac death, MI, and stroke from all available trials were included sequentially according to the year in which they first became available.

We explored potential sources of heterogeneity in estimates of the treatment effect on MACE with univariate meta-regression [50] (for number of participants, mean age, percentage of men, baseline homocysteine level, baseline folate level, dose of folic acid, dose of vitamin B6, dose of vitamin B12, net decrease in homocysteine level, and duration of follow-up). Subsequently, subgroup analyses were conducted for MACE on the basis of number of participants, mean age, percentage of men, number of trial centers, baseline homocysteine level, baseline folate level, intervention regimens, dose of folic acid, dose of vitamin B6, dose of vitamin B12, net decrease in homocysteine level, background fortification, control, disease prevention, renal status, duration of follow-up, and Jadad score [45]. Interaction tests [51] were performed to compare differences between estimates of the 2 subsets, which were based on Student *t* distribution rather than on normal distribution because the number of included studies was small. We also performed a sensitivity analysis by removing each individual trial from the meta-analysis [52]. Several methods were used to check for potential publication bias. Visual inspection of funnel plots for MACE, total mortality, cardiac death, MI, and stroke were conducted. The Egger (53) and Begg test (54) were used to statistically assess publication bias for MACE, total mortality, cardiac death, MI, and stroke. All reported P values were two-sided, and P values of <0.05 were considered statistically significant for all included studies. Statistical analyses were performed using STATA software (version 10.0 StataCorp, Texas, USA).

## Results

### Search of the Published Literature

**Figure 1:**
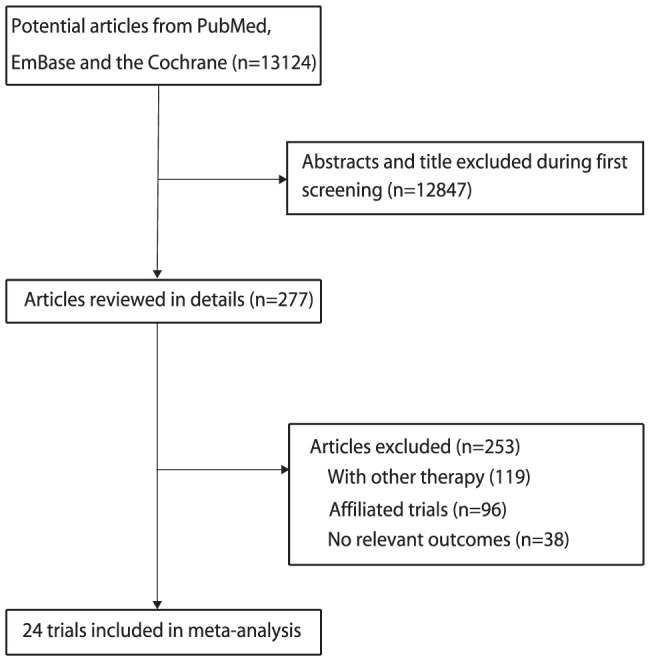
Flow diagram of the literature search and trials selection process.

We identified 13,124 articles during our initial electronic search, of which 12,847 were excluded during an initial review of the titles and abstracts. We retrieved the full text for the remaining 277 articles, and 24 RCTs [20]–[43] met the inclusion criteria of our meta-analysis (**Figure 1**). B vitamin supplementation for primary prevention of major cardiovascular outcome was studied in 12 trials [32]–[43], and the remaining 12 trials studied its use for secondary prevention [20]–[31].

### Characteristics of the Included Studies

**Tables 1** **and S1** summarizes the baseline characteristics of the 57,952 individuals included in the trials investigated. The follow-up period for participants ranged from 0.7 to 7.3 years, the number of individuals included in each trial ranged from 81 to 12064, and the net change in total homocysteine level ranged from 1.3 to 26.0 µmol/L. In the intervention groups, the dose of folic acid ranged from 0.5 to 40 mg/day, the dose of vitamin B6 ranged from 3 to 250 mg/day, and the dose of vitamin B12 ranged from 6 to 2000 µg/day. In the meta-analysis, the trial outcome was MACE in 23 trials [20]–[36], [38]–[43], total mortality in 20 trials [21]–[31], [33], [35], [37]–[43], cardiac death in 15 trials [21]–[23], [25]–[27], [29]–[31], [34]–[36], [39], [42], [43], MI in 19 trials [21]–[31], [33], [35], [37]–[39], [41]–[43], and stroke in 18 trials [22], [23], [25]–[31], [33]–[35], [37]–[39], [41]–[43]. We restricted the inclusion criteria to RCTs with a minimum follow-up of 6 months to ensure a reliable conclusion.

**Table 1:** Design and characteristic of trials included in our meta-analysis*.

Source	Publication year	No. of patients	Mean age, y	Percentage male (%)	Background fortification	Disease status	Primary/Secondary prevention	Baseline folate status (nmol/L)	Baseline homocysteine (µmol/L)	Net decrease in homocysteine (µmol/L)	Intervention	Follow-up (year)	Jadad score
Baker F(20)	2002	1882	NG	NG	No	CHD	Secondary	NG	11.2	−1.5	5.0 mg folic acid; placebo	1.7	1
The Swiss Heart Study(21)	2002	553	63	80	No	Coronary angioplasty	Secondary	NG	11.2	−2.9	1.0 mg folic acid, 10 mg vitamin B6, and 0.4 mg vitamin B12; Placebo	1.0	3
M Righetti(32)	2003	81	64	56	No	ESRD	Primary	6.06	50.3	−26.0	25 mg folic acid; 5 mg folic acid; untreated	1.0	1
VISP Trial Investigators(22)	2004	3680	66	63	Yes	Ischemic stroke	Secondary	NG	12.3	−2.1	25 mg of vitamin B6, 0.4 mg of vitamin B12, and 2.5 mg of folic acid; 200 µg of vitamin B6, 6 µg of vitamin B12, and 20 µg of folic acid	2.0	5
A Liem(23)	2004	283	59	69	No	CHD	Secondary	NG	NG	NG	5 mg folic acid; placebo	1.0	2
EM Wrone(33)	2004	510	60	50	Yes	ESRD	Primary	47.07	32.9	−3.6	15 mg folic acid, 12.5 mg vitamin B6, 6 µg vitamin B12; 5 mg folic acid, 12.5 mg vitamin B6, 6 µg vitamin B12; 1 mg folic acid, 12.5 mg vitamin B6, 6 µg vitamin B12	2.0	4
H Lange(24)	2004	636	61	77	No	Coronary Stenting	Secondary	NG	12.6	−3.6	1.2 mg of folic acid, 48 mg of vitamin B6,and 60 µg of vitamin B12; placebo	0.7	3
A Liem(25)	2005	593	65	78	No	CHD	Secondary	16	12.1	−1.3	0.5 mg folic acid; usual care	3.2	3
NORVIT Trial Investigators(26)	2006	3749	63	74	No	MI	Secondary	10.95	13.1	−2.3	0.8 mg of folic acid, 0.4 mg of vitamin B12, and 40 mg of vitamin B6; placebo	3.3	5
(HOPE) 2 Investigators(27)	2006	5522	69	72	Parial	DM or vascular disease	Secondary	28.0	12.2	−3.3	2.5 mg of folic acid, 50 mg of vitamin B6, and 1 mg of vitamin B12; placebo	5.0	4
ASFAST Study Group(34)	2006	315	56	68	Yes	ESRD	Primary	NG	27.0	−4.7	15 mg folic acid; placebo	3.6	4
M Righetti(35)	2006	114	64	55	No	Hemodialysis	Primary	22.32	31.7	−15.1	5 mg folic acid plus vitamin B1 250 mg, vitamin B6 250 mg, vitamin B12 500 µg; untreated	2.4	2
ACA Vianna(36)	2007	186	48	59	No	ESRD	Primary	9.99	24.6	−10.5	Folic acid 10 mg 3 times a week; placebo	2.0	2
Polyp Prevention Study Group(37)	2007	1021	57	64	Yes	Colorectal adenomas	Primary	23.70	9.8	NG	1 mg/d of folic acid daily; placebo	7.0	4
Veterans Affairs Site Investigators(38)	2007	2056	66	98	Yes	CKD or ESRD and high tHcy	Primary	35.34	22.4	−5.1	40 mg of folic acid, 100 mg of vitamin B6, and 2 mg of vitaminB12; placebo	3.2	4
WAFACS Study Group(39)	2008	5442	63	0	No	Health professionals	Primary	NG	NG	NG	2.5 mg of folic acid, 50 mg of vitamin B6, and 1 mg of vitamin B12; placebo	7.3	3
WENBIT Study Group(28)	2008	3096	62	80	No	Coronary angiography	Secondary	NG	11.1	−2.8	Folic acid, 0.8 mg, plus vitamin B12, 0.4 mg, plus vitamin B6, 40 mg; folic acid plus vitamin B12; vitaminB6 alone; placebo	3.1	4
BVAIT Research Group(40)	2009	506	61	61	Yes	Initial tHcy>8.5 umol/L	Primary	21.41	9.6	−2.1	5 mg folic acid, 0.4 mg vitamin B12 plus 50 mg vitamin B6; placebo	3.1	3
DIVINe Study Group(41)	2010	238	60	75	Yes	Diabetic nephropathy	Primary	35.12	15.6	−4.8	2.5 mg folic acid, 25 mg vitamin B6, and 1 mg vitamin B12; placebo	3.0	4
J Heinz(42)	2010	650	61	58	No	ESRD	Primary	14.1	29.0	−8.6	2.5 mg folic acid, 25 µg vitamin B12, and 10 mg vitamin B6; 0.1 mg folic acid, 2 µg vitamin B12, and 0.5 mg vitamin B6	2.1	5
SEARCH Collaborative Group(29)	2010	12064	64	83	No	MI	Secondary	16.76	13.5	−3.8	2 mg folic acid plus 1 mg vitamin B12 daily; placebo	6.7	4
SU.FOL.OM3 Collaborative Group(30)	2010	2501	61	79	No	MI, angina, or ischaemic stroke	Secondary	15.29	12.8	−2.7	5-methyltetrahydrofolate (560 µg), vitamin B-6 (3 mg), and vitamin B-12 (20 µg); placebo	4.7	5
FAVORIT Study Group(43)	2011	4110	52	63	Yes	Kidney transplant recipients	Primary	NG	16.4	−4.1	5.0 mg folic acid, 50 mg vitamin B6, and 1.0 mg vitamin B12; 1.4 mg vitamin B6 and 0.002 mg vitamin B12	4.0	4
VITATOPS Study Group(31)	2012	8164	63	64	Partial	TIA or stroke	Secondary	NG	14.3	−3.8	2 mg folic acid, 25 mg vitamin B6, and 0·5 mg vitamin B12; placebo	3.4	5

*CHD: coronary heart disease; ESRD: End-stage renal disease; MI: myocardial infarction; DM: diabetes mellitus; CKD: chronic kidney disease; TIA: transient ischaemic attack; NG: not give.

### Risk of Bias in Individual Trials

The quality of the trials was assessed using the Jadad score (45) and are summarized in **Table 1**. We considered a score ≥4 to indicate a high-quality study. Five trials [22], [26], [30], [31], [42] had a Jadad score of 5, 9 trials [27]–[29], [33], [34], [37], [38], [41], [43] scored 4, 5 trials [21], [24], [35], [39], [40] scored 3, 3 trials [23], [35], [36] scored 2, and the remaining 2 trials [20], [32] scored 1.

### Effects of B Vitamins on Major Cardiovascular Outcomes

**Figure 2:**
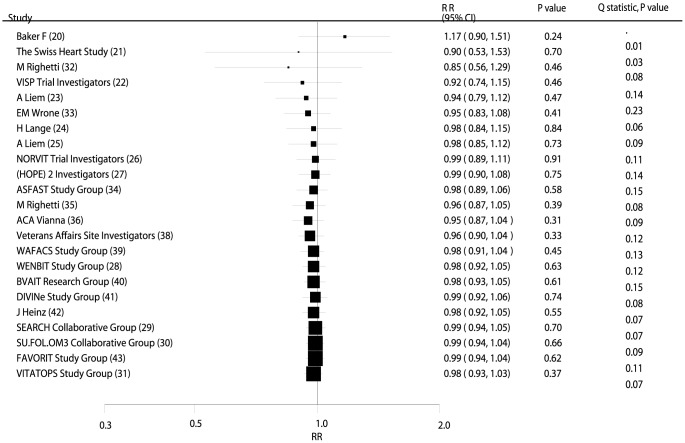
Cumulative meta-analysis of the B vitamins supplementation for major adverse cardiovascular event. RR, relative risk; CI, confidence interval.

Data from 56,925 individuals was used to assess the effect of B vitamin supplementation on MACE and included 10,917 MACE. Overall, B vitamin supplementation reduced the risk of MACE by 2%, but this was not statistically significant (RR, 0.98; 95% CI: 0.93–1.03; P = 0.37, **Figure 2** and **Figure S1**). Heterogeneity was observed in the magnitude of the effect across the trials (P = 0.07). However, after sequential exclusion of each trial from all pooled analysis, the conclusion was not affected by the exclusion of any specific trial.

**Figure 3:**
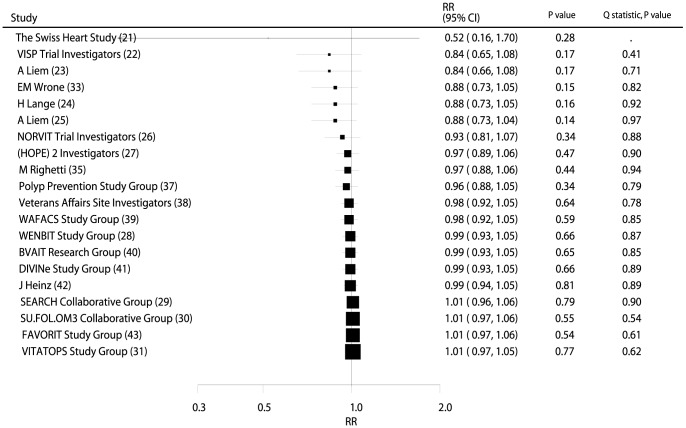
Cumulative meta-analysis of the B vitamins supplementation for total mortality. RR, relative risk; CI, confidence interval.

Data from 55,482 individuals was used to assess the effect of B vitamin supplementation on total mortality and included 7,203 deaths. No significant difference in the number of deaths was observed between participants receiving B vitamins compared with those receiving placebo (RR, 1.01; 95% CI: 0.97–1.05; P = 0.77, without evidence of heterogeneity; **Figure 3** and **Figure S2**).

**Figure 4:**
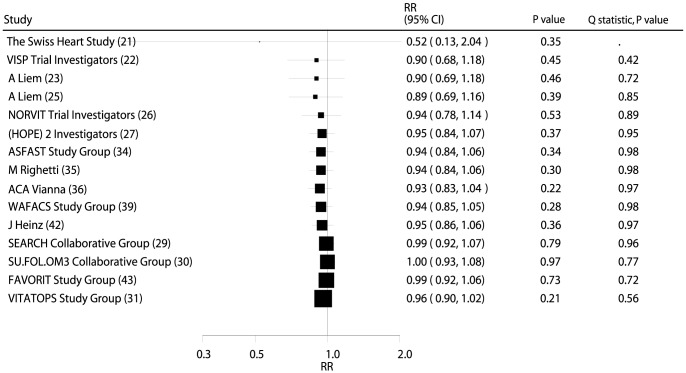
Cumulative meta-analysis of the B vitamins supplementation for cardiac death. RR, relative risk; CI, confidence interval.

Data from 47,926 individuals was used to assess the effect of B vitamin supplementation on cardiac death and included 3,422 cardiac deaths. B vitamin supplementation caused a 4% reduction in cardiac death; however, this was not a significant change (RR, 0.96; 95% CI: 0.90–1.02; P = 0.21, without evidence of heterogeneity; **Figure 4** and **Figure S3**).

**Figure 5:**
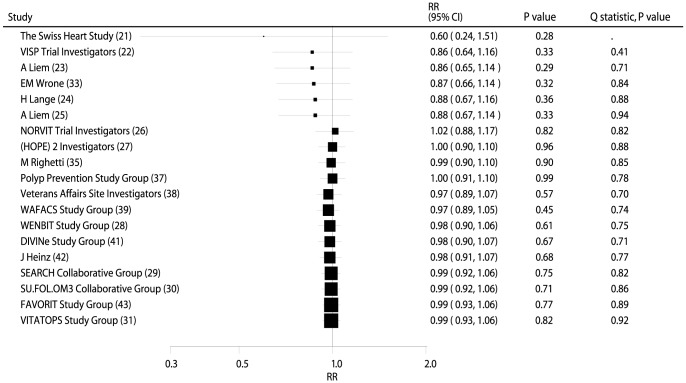
Cumulative meta-analysis of the B vitamins supplementation for myocardial infarction. RR, relative risk; CI, confidence interval.

Data from 54,976 individuals was used to assess the effect of B vitamin supplementation on MI, and included 3,623 MIs. There were no significant differences between participants receiving B vitamins compared with placebo for MI (RR, 0.99; 95% CI: 0.93–1.06; P = 0.82, without evidence of heterogeneity, **Figure 5** and **Figure S4**).

**Figure 6:**
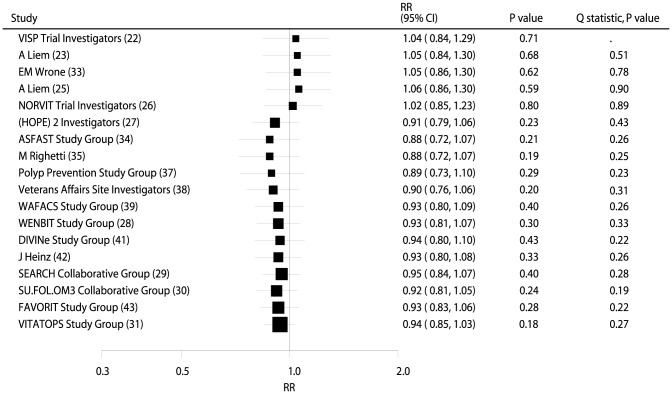
Cumulative meta-analysis of the B vitamins supplementation for stroke. RR, relative risk; CI, confidence interval.

Data from 54,102 individuals and 2,465 stroke events was used to assess the effect of B vitamin supplementation on stroke. B vitamin supplementation reduced incident stroke by 6%, but this was not a significant reduction (RR, 0.94; 95% CI: 0.85–1.03; P = 0.18; with no statistical heterogeneity, **Figure 6** and **Figure S5**). Sensitivity analysis was conducted for the incidence of stroke. However, after sequential exclusion of each trial from all pooled analysis, the conclusion was not affected by the exclusion of any specific trial.

### Cumulative Meta-Analysis

On cumulative meta-analysis for MACE (**Figure 2**), the original nonsignificant result for a B vitamin effect persisted, and the effect was slight and borderline nonsignificant. Similarly, the nonsignificant result persisted when cumulative meta-analyses for total mortality (**Figure 3**), cardiac death (**Figure 4**), MI (**Figure 5**), and stroke (**Figure 6**) were conducted.

### Meta-Regression and Subgroup Analyses

Heterogeneity testing for the analysis showed a P<0.10 for MACE. We concluded that heterogeneity was statistically significant in the overall analysis. We, therefore, conducted a meta-regression analysis [50] for MACE that included the number of participants, mean age, percentage of men, baseline homocysteine level, baseline folate level, dose of folic acid, dose of vitamin B6, dose of vitamin B12, net decrease in homocysteine level, and duration of follow-up. Overall, we detected a net decrease in homocysteine (P = 0.043) that contributed to the association between B vitamin supplementation and MACE (**Figure S6**). However, the number of participants (P = 0.435), mean age (P = 0.763), percentage of men (P = 0.866), baseline homocysteine level (P = 0.094), baseline folate level (P = 0.797), dose of folic acid (P = 0.840), dose of vitamin B6 (P = 0.933), dose of vitamin B12 (P = 0.614), and duration of follow-up (P = 0.186) were not significant factors contributing to the association between B vitamin supplementation and MACE (**Figure S6**).

**Figure 7:**
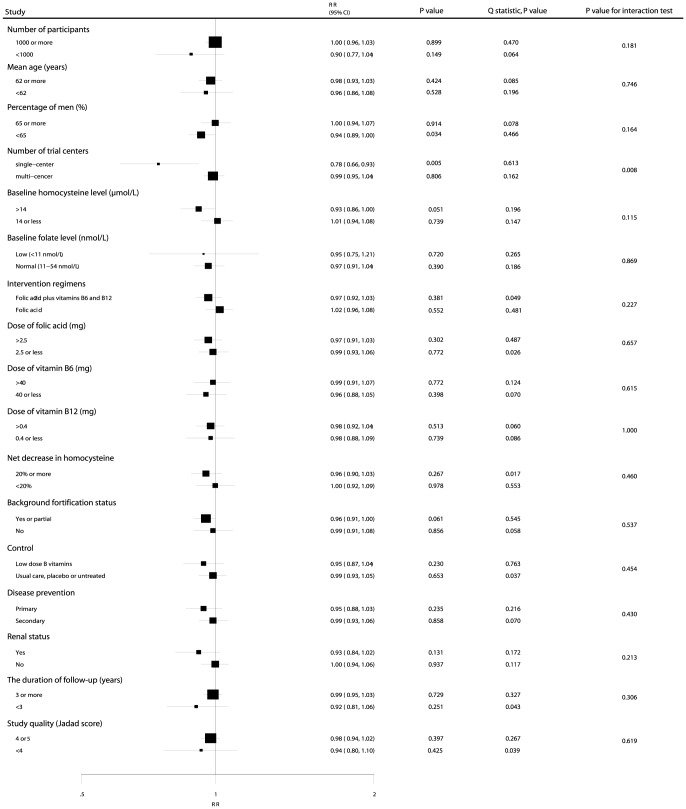
Subgroup analysis for the effect of B vitamins supplementation on major cardiovascular events. RR, relative risk; CI, confidence interval.

Subgroup analyses were conducted for MACE to minimize heterogeneity among the included trials and to evaluate the effect of B vitamin supplementation in specific subpopulations. B vitamin supplementation significantly reduced the risk of MACE if the percentage of men in the study was <65% (RR, 0.94; 95% CI: 0.89–1.00; P = 0.034; **Figure 7**) and the study was a single-center trial (RR, 0.78; 95% CI: 0.66–0.93; P = 0.005; **Figure 7**). Furthermore, we noted that B vitamin supplementation was associated with a nonsignificant reduction in MACE when the baseline homocysteine level was>14 µmol/L (RR, 0.93; 95% CI: 0.86–1.00; P = 0.051; **Figure 7**) or dietary grain fortification had been taken to boost the folate level (RR, 0.96; 95% CI: 0.91–1.00; P = 0.061; **Figure 7**). No other significant differences were identified in the predefined factors between those who took B vitamin supplements and those who took placebo (**Figure 7**). Furthermore, there was no significant difference in the effect of B vitamins on MACE between the 2 subgroups by factors that could affect the treatment effect except center of trials (P = 0.008; **Figure 7**).

### Publication Bias

Review of funnel plots did not rule out the potential for publication bias for MACE, total mortality, cardiac death, MI, and stroke (**Figure S7**). However, the Egger [53] and Begg tests [54] showed no evidence of publication bias for MACE (P value for Egger, 0.342; P value for Begg, 0.267), total mortality (P value for Egger, 0.312; P value for Begg, 0.183), cardiac death (P value for Egger, 0.631; P value for Begg, 0.621), MI (P value for Egger, 0.816; P value for Begg, 0.944), and stroke (P value for Egger, 0.992; P value for Begg, 1.000).

## Discussion

Several observational studies [1]–[5] have suggested that B vitamin supplementation may improve major cardiovascular outcomes. However, this effect has not been confirmed to date. In the present study, we included RCTs and explored all possible correlations between B vitamin supplementation and the outcomes of MACE, total mortality, cardiac death, MI, and stroke. Furthermore, we conducted a cumulative meta-analysis to explore the value of routine B vitamin supplementation in clinical practice. This large quantitative study included 57,952 individuals from 24 trials with a broad range of baseline characteristics. Our results suggest that B vitamin supplementation has no significant effect on MACE, total mortality, cardiac death, MI, or stroke. Furthermore, in a cumulative meta-analysis, this B vitamin effect persisted and remained nonsignificant.

We reviewed previous meta-analyses and found that the hypothesized protective effect of B vitamins comes from observational studies [9], which, we suggest, may overestimate the effect on major cardiovascular outcomes. Several systematic reviews and meta-analyses of RCTs [12]–[17] have evaluated the impact of folic acid supplementation on major cardiovascular outcomes and have found no evidence to support a significant association. In a meta-analysis, Wang et al. [19] indicated that B vitamins significantly reduced the risk of stroke, but subgroup analysis showed that this effect was based on trials that included only participants without a previous stroke history. Lee et al. [55] suggested that folic acid supplementation administered as primary prevention significantly reduced the risk of stroke at>3 years of follow-up and that the homocysteine-lowering effect was>20%. However, this meta-analysis included trials with a male: female ratio of >2. Gao et al. [56] found that B vitamin supplementation given to reduce homocysteine levels influenced the risk of stroke events, especially in subgroups with a follow-up of>3 years (RR, 0.92; 95% CI: 0.84–1.00), without a history of grain fortification (RR, 0.91; 95% CI: 0.83–1.00), or without chronic kidney disease (RR, 0.93; 95% CI: 0.86–1.01), but these differences were not significant. In the present study, all pooled RR estimate points for stroke were <1 (evidence accumulated up to 2006) with a potential trend toward moving leftward in the cumulative meta-analysis of B vitamin supplementation. We suggest a potential protective effect of B vitamins on stroke events. However, this trend is not obvious and requires validation.

There was no significant difference between B vitamin supplementation and placebo for the RR of major cardiovascular outcomes. However, several trials included in our study reported inconsistent results. Schnyder et al. [21] indicated that homocysteine-lowering therapy with B complex vitamins reduced the risk of MACE in patients after percutaneous coronary intervention. However, Lange et al. [24] indicated that B vitamin complex supplementation after coronary stenting increases the risk of in-stent restenosis and the need for target-vessel revascularization. The possible reasons for these inconsistencies are as follows: (1) a history of B vitamin supplementation may mask treatment effect; (2) patients in our meta-analysis had variable disease status, which also affects major cardiovascular outcomes; (3) elevated homocysteine levels impair vascular function, but the effect of homocysteine on cardiovascular outcomes may not be directly related to coronary disease pathogenesis, which is largely attributable to plaque formation and rupture [57]; and (4) high doses of B vitamin may adversely affect vascular remodeling and myocardial repair and may increase complications and death in patients with cardiovascular disease [58]. Therefore, although B vitamin supplementation may have direct effects on major cardiovascular outcomes, these effects may be counterbalanced by other effects.

Cumulative meta-analyses suggest that a nonsignificant response persisted for MACE, total mortality, cardiac death, MI, and stroke. However, the proposed nonsignificant protective effect of B vitamin supplementation for MI has been refuted by the NORVIT Trial Investigators [26], who specifically included individuals with MI, which may have contributed to higher recurrence and suggests a nonsignificant harmful effect for B vitamin supplementation. However, the proposed nonsignificant harmful effect of B vitamin supplementation for stroke was refuted by the HOPE 2 Trial [27]. This trial suggested that B vitamin supplementation reduced the risk of stroke by 25% (95% CI: 0.59–0.97), but this may be due to a considerably lower number of stroke events than coronary events. In addition, the CIs of the estimated risk reduction were wide. Furthermore, the results were not adjusted for the multiplicity of potential outcomes.

In our study, subgroup analysis suggested that B vitamin supplementation reduced the risk of MACE in specific subgroups. The main findings were as follows. First, although no significant difference was observed in MACE for the B vitamin supplementation group compared with the placebo group, in populations with a baseline total homocysteine level >14 µmol/L and those with a net decrease in homocysteine level of >20% there seemed to be a slight, but nonsignificant, benefit. In this study, baseline homocysteine levels were only available for whole populations, not individuals. Second, folate fortification of the grain supply may influence the risk of MACE, because it reduces the prevalence of low folate levels and high total homocysteine levels, so the mean difference in total homocysteine levels between groups narrows. Fortification reduces the number of participants with a high total homocysteine level, which is the population most likely to benefit from supplementation [59]. In this study, we found that B vitamin supplementation reduced the risk of MACE if participants had a background of grain fortification. Those with background fortification received higher doses of vitamin B12, which may be more effective at preventing MACE. Third, the protective effect of B vitamins was more evident if the percentage of men in the study was <65%. Among men, there is a higher rate of smoking and drinking, both of which increase the risk of MACE. Therefore, the effect of B vitamins on the risk of MACE in studies with a higher percentage of male subjects may be reduced or balanced by a higher rate of smoking and drinking. Fourth, there was a significant difference between single-center and multi-center trials in the risk for MACE. This difference could be due to chance because only 6 trials were single-center, and they had a low sample size and low rate of cardiovascular disease occurrence. Fifth, no significant differences were observed in MACE between participants with a low folate level and those with a normal folate level. Folate level data were not available for several trials, so this conclusion was similar to those of a previous meta-analysis [60]. Sixth, the B vitamin dose showed no significant effect because all doses investigated were in the mega-dose range, and there may have been differences in the control groups in different studies. Seventh, B vitamin supplementation seems to offer a benefit for primary prevention of MACE but not for secondary prevention. Participants with pre-existing cardiovascular disease have higher recurrence and mortality rates. Finally, B vitamins seem to benefit participants with kidney disease but not those without kidney disease; patients with kidney disease may have higher homocysteine concentrations and a higher rate of cardiovascular disease.

The limitations of our study are as follows: (1) different types and doses of supplements might result in bias; (2) the use of background B vitamin supplementation might have impaired our ability to identify a treatment effect; (3) differences in diagnosis and reporting may have contributed to the differences in major cardiovascular outcomes in some trials; (4) stratified analyses based on background therapy in patients with previous disease were unavailable; (5) data on hemoglobin levels were not available, so we could not evaluate the potential confounding role of hemoglobin when evaluating the effect of vitamins B on the risk of major cardiovascular outcomes; (6) several trials with low Jadad score were included in our study, which could bias the results; and (7) since inherent assumptions are made for any meta-analysis, the analysis used pooled data, and individual patient data were unavailable; this restricted us from performing a more detailed analysis and obtaining more comprehensive results.

In conclusion, we found that B vitamin supplementation has little or no effect on major cardiovascular outcomes across various patient populations. We believe the use of B vitamin supplementation as a structured intervention in everyday clinical practice is not justified. A meta-analysis of individual patient data might be more appropriate to determine how the duration and dose of supplementation influence treatment outcomes.

## Supporting Information

Figure S1 **Effect of B vitamins supplementation on the risk of major cardiovascular events.**
(EPS)

Figure S2 **Effect of B vitamins supplementation on the risk of total mortality.**
(EPS)

Figure S3 **Effect of B vitamins supplementation on the risk of cardiac death.**
(EPS)

Figure S4 **Effect of B vitamins supplementation on the risk of myocardial infarction.**
(EPS)

Figure S5 **Effect of B vitamins supplementation on the risk of stroke.**
(EPS)

Figure S6 **Meta-regression of number of participants, mean age, percentage male, baseline homocyteine, baseline folate status, dose of folic acid, dose of vitamin B6, dose of vitamin B12, net decrease in homocysteine, and the duration of the follow-up periods.**
(EPS)

Figure S7 **Funnel plots for major cardiovascular events, total mortality, cardiac death, myocardial infarction, and stroke.**
(EPS)

Table S1 **Additional characteristic of trials included in our meta-analysis.**
(DOC)

Checklist S1 **PRISMA Checklist.**
(DOC)
