# Supplementary material for: Association between B Vitamins Supplementation and Risk of Cardiovascular Outcomes: A Cumulative Meta-Analysis of Randomized Controlled Trials
Source: PLoS One. 2014 Sep 19;9(9):e107060. doi: 10.1371/journal.pone.0107060 (PMC4169527; doi:10.1371/journal.pone.0107060)
Supplement: Table S1 — Additional characteristic of trials included in our meta-analysis. (DOC) [file pone.0107060.s008.doc]

Table S1. Additional characteristic of trials included in our meta-analysis

| Study | Center of trials | Antiplatelet agents (%) | Lipid-lowering agents (%) | Oral anticoagulants (%) | ACE inhibitors/angiotensin II blockers (%) |
| --- | --- | --- | --- | --- | --- |
| Baker F(20) | Multi-center | - | - | - | - |
| The Swiss Heart Study(21) | Single-center | 93/94 | 69/71 | - | 37/36 |
| M Righetti(32) | Single-center | - | - | - | - |
| VISP Trial Investigators(22) | Multi-center | - | - | - | - |
| A Liem(23) | Single-center | 97/99 | - | - | 36/39 |
| EM Wrone(33) | Multi-center | - | - | - | - |
| H Lange(24) | Multi-center | - | 37.7/41.6 | - | 28.5/23.8 |
| A Liem(25) | Single-center | - | - | - | - |
| NORVIT Trial Investigators(26) | Multi-center | 89/88 | 81/81 | 11/12 | 31/30 |
| (HOPE) 2 Investigators(27) | Multi-center | 77.9/80.5 | 59.0/61.1 | - | 65.9/66.1 |
| ASFAST Study Group(34) | Multi-center | 21.8/31.4 | 28.8/28.9 | 5.1/6.9 | 26.9/25.2 |
| M Righetti(35) | Single-center | - | - | - | - |
| ACA Vianna(36) | Single-center | - | - | - | - |
| Polyp Prevention Study Group(37) | Multi-center | - | - | - | - |
| Veterans Affairs Site Investigators(38) | Multi-center | 45/40 | 50/48 | - | 42/39 |
| WAFACS Study Group(39) | Multi-center | - | 33.6/34.5 | - | 24.3/25.8 |
| WENBIT Study Group(28) | Multi-center | 90.0/90.9 | 88.3/88.6 | 5.2/4.1 | 31.2/35.0 |
| BVAIT Research Group(40) | Multi-center | - | 15.4/14.3 | - | 27.2/29.4 |
| DIVINe Study Group(41) | Multi-center | 62.2/62.2 | 77.3/79.0 | - | 56.3/62.2 |
| J Heinz(42) | Multi-center | - | - | - | - |
| SEARCH Collaborative Group(29) | Multi-center | - | 100/100 | - | 42/42 |
| SU.FOL.OM3 Collaborative Group(30) | Multi-center | 92.9/94.0 | 84.5/86.4 | - | 53.8/53.5 |
| FAVORIT Study Group(43) | Multi-center | - | - | - | - |
| VITATOPS Study Group(31) | Multi-center | - | - | - | - |
